# Supplementary figures and images for: Adaptations to nitrogen availability drive ecological divergence of chemosynthetic symbionts
Source: PLoS Genet. 2024 May 31;20(5):e1011295. doi: 10.1371/journal.pgen.1011295 (PMC11168628; doi:10.1371/journal.pgen.1011295)

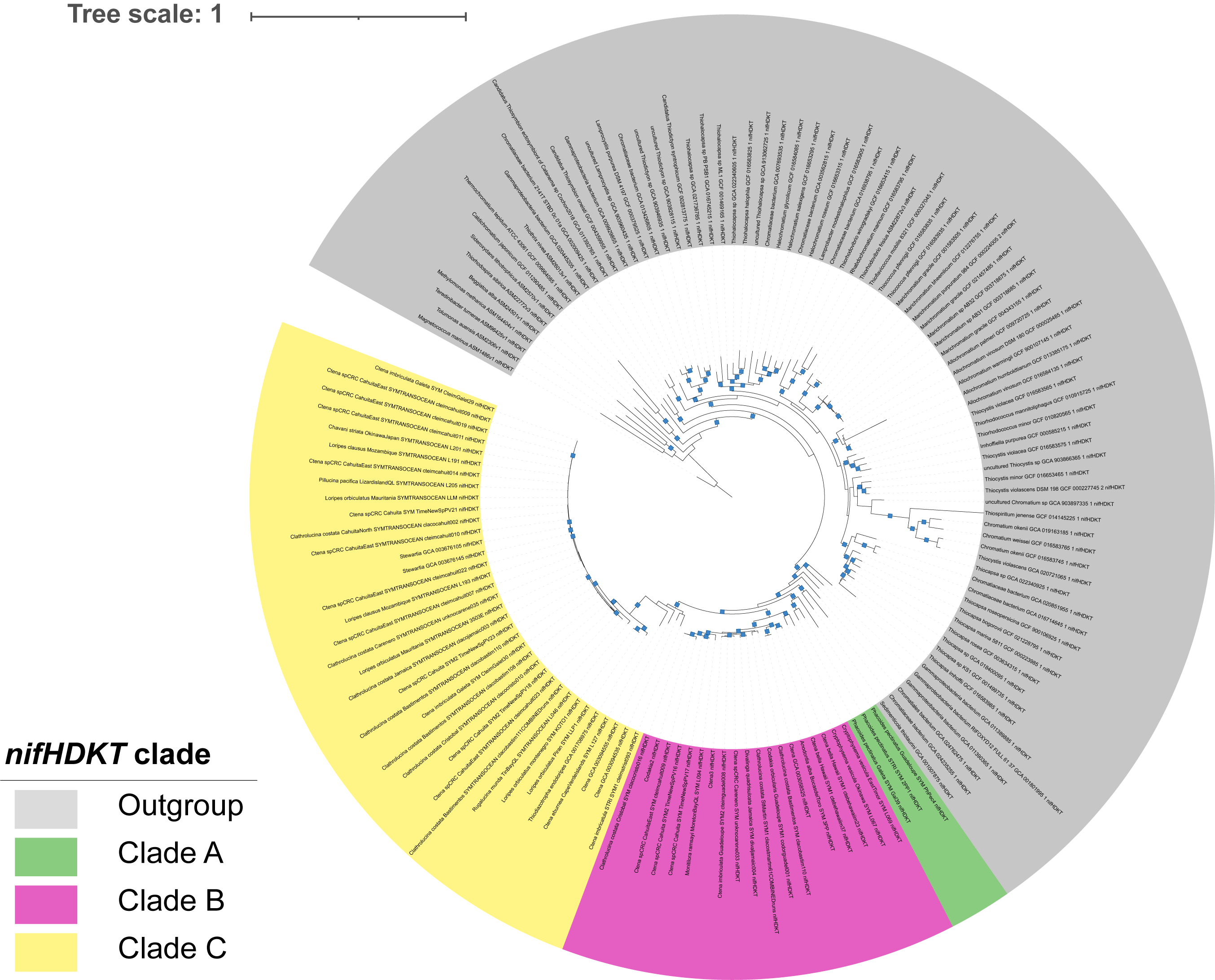

Supplement: S2 Fig — (TIF) [file pgen.1011295.s010.tif]

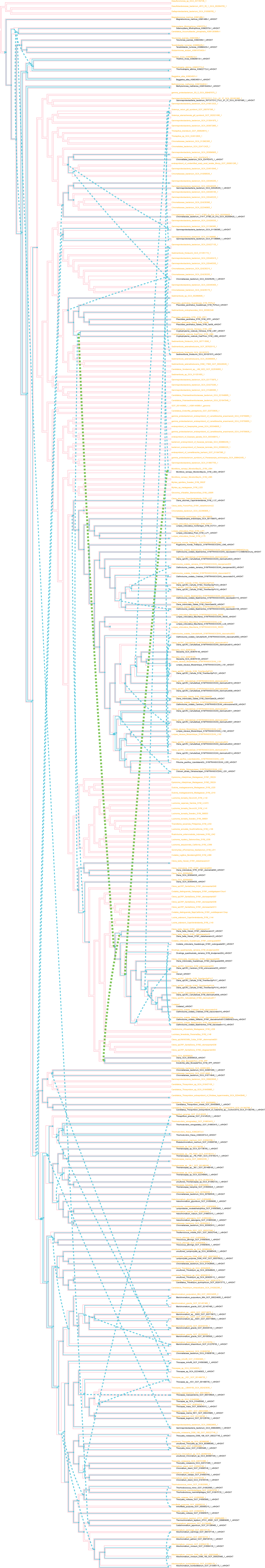

Supplement: S3 Fig — (TIF) [file pgen.1011295.s011.tif]

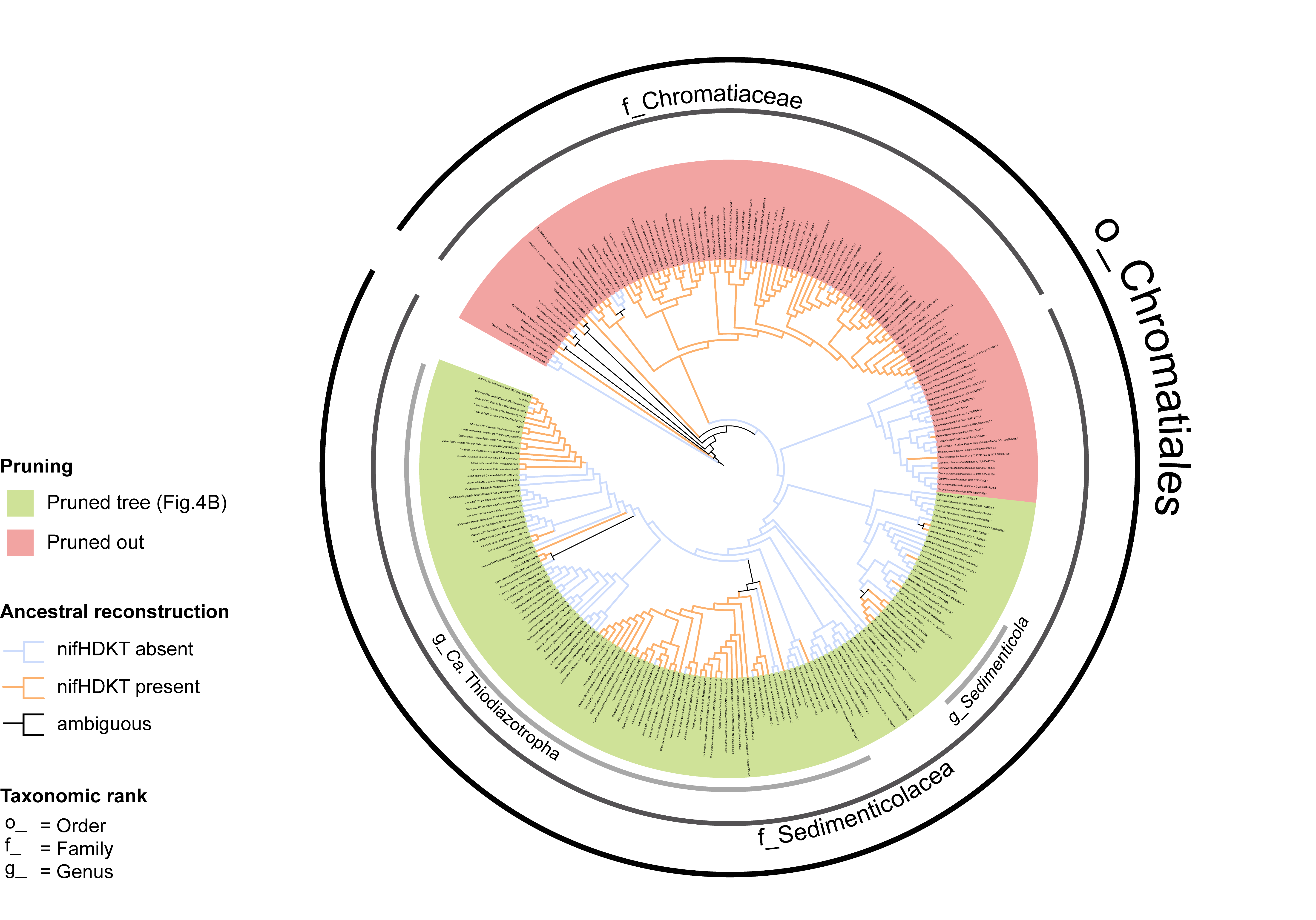

Supplement: S4 Fig — (TIF) [file pgen.1011295.s012.tif]

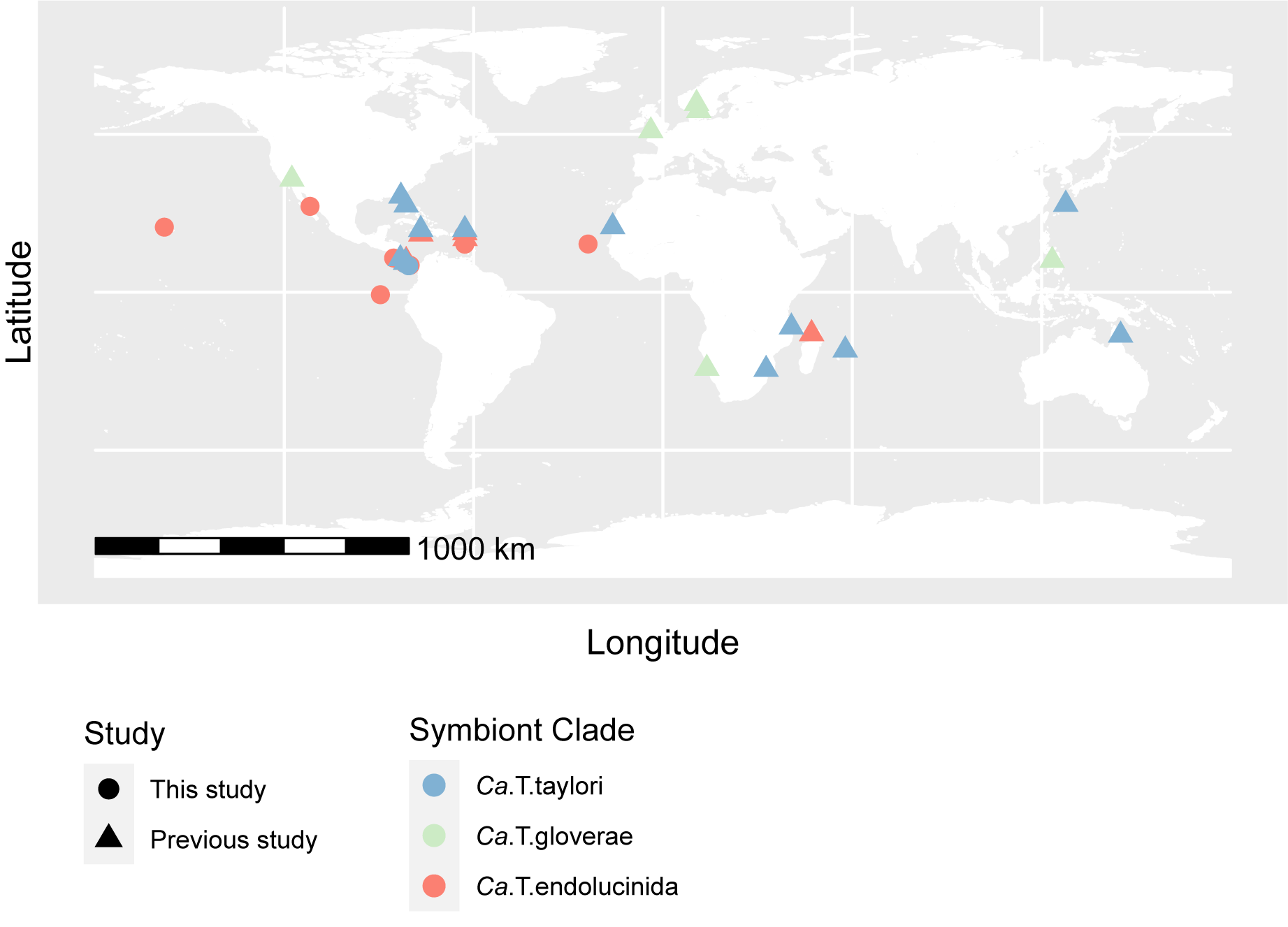

Supplement: S5 Fig — The map was generated with data from Natural Earth (http://www.naturalearthdata.com/) using the R package "rnaturalearth" (v0.3.2) (https://github.com/ropensci/rnaturalearth) (TIF) [file pgen.1011295.s013.tif]
