# Supplementary material for: Adaptations to nitrogen availability drive ecological divergence of chemosynthetic symbionts
Source: PLoS Genet. 2024 May 31;20(5):e1011295. doi: 10.1371/journal.pgen.1011295 (PMC11168628; doi:10.1371/journal.pgen.1011295)
Supplement: Attachment — Submitted filename: 240108_Morel-Letelier_PLoSGen.pdf [file pgen.1011295.s014.pdf]

Overview:
Beautiful work showing horizontal gene transfer in an endosymbiosis that underwent dramatic divergence when the isthmus of Panama formed. The host speciated due to vicariance and the endosymbiont on the oligotrophic Caribbean side was swapped for a N-fixing strain that had acquired its N-fixing genes by HGT prior to the association.

I think this work is important and well-done. However, I have a few concerns about the completeness of the assemblies, given the reliance of many claims on “gene absence”. Hopefully most of these concerns can be addressed by more transparent presentations of assembly completeness and coverage (as described below). I am also concerned about the functional claims that are made given that these are genome assembly-based predictions and no nitrogen-fixation, or other physiological, data were obtained or cited. Lastly, I think the authors could have done more due diligence in resolving the ancestral state of the Ca. Thiodiazotropha + Sedimenticola most recent common ancestor. Overall, I think these issues can be addressed in a short time frame, so I recommend major revisions.

Minor Comments:
Figure 1D: the top axis labels are flip-flopped (symbiont clade = purple or green / host genus = violet or pink).

Figure 3B: Since many of your claims rely on ecology and geography it would help the reader to have the other samples in the tree (that you didn’t collect/sequence) color coded by geography.

Line 443: typo in the word “closely” - “*the Pegophyseminae symbionts “PEGO” or an unsampled closel related lineage*.”

Lines 479-480: “*This suggests there is no barrier to gene flow between geographically separated populations of Ca. T. endolucinda*”

> There might be some barriers that could be quantified with a proper admixture demographic analysis. It is safer (in terms of accuracy) to say “low barriers to gene flow”.

Lines 548-549: “*Samples ctemesantah004, cteimcahuit014, and cteimbast119 were also sequenced using PacBio Sequel II long-read technology.*”

> Why only these samples? I can’t find an explanation for the choice anywhere in the manuscript.

Major Comments:

Line 610: “*ANI values were calculated with fastANI v1.1*”

> Are all ANI values based on genome-wide comparisons? Did you only include “core” regions? Please state what ANI was calculated from in the text (I didn’t see it elsewhere either).

Figure 1 legend (line 188) and Figure 2 legend (line 300): Why was the plant protein model matrix (Q.plant+F+I+G4) used for bacterial symbiont tree inference? See http://www.iqtree.org/doc/Substitution-Models.

So many of your functional hypotheses rely on the absence of particular genes (Figures 2-4). However, I don’t have a good sense for how complete these MAGs are by the time you start discussing the lack of form II rubisco in Ca. T. fergusoni on lines 224-225 and the absence of nitrogen pathways in TEP MAGs on lines 231-234. Annotated genes may be missing due to broken, incomplete assemblies. You have a very clear signal

across samples in Figure 2, so I think this signal is not due to some technical artifact. But it would help the reader have confidence in your subsequent claims to present coverage and completeness statistics upfront.

> You do seem to try to account for an ascertainment bias - that the TEP nitrogen genes weren't annotated - when you state (on lines 245-247): "no hits were reported from the hmmer search search of the Pfam nitrogenase model against the TEP metagenome assemblies, supporting the lack of nitrogen fixation potential in the TEP symbiont genomes". But, what about in the unassembled reads? Were these assemblies more fragmented than the Caribbean MAGs? If so, maybe the nitrogen genes were in the regions that could not assemble. I need some evidence that this is not the case.
>
> Layering assembly quality information on Table 1 could help contextualize the patterns of incomplete presence/absence. I.e., are those near, but not total fixations for some KOfam terms among Caribbean MAGs due to the same samples, and did those samples produce worse assemblies?
>
> Lines 625-628: "*To confirm the lack of nitrogen fixation potential in TEP symbionts' genomes and ensure that the genes were not missed in the binning process, the proteins were directly predicted from all TEP metagenome assemblies with Prodigal v2.6.3 [80].*"
>
> > This will still miss genes that are on unassembled or fragmented contigs. Attempting to "fish" the genes out by read mapping would enable you to look for these genes with less assembly-based bias.

Lines 459-462: Indeed, I was thinking that including a few more divergent outgroup taxa could enable you to determine the nif content state for the last common ancestor of the Ca. Thiodiazotropha and Sedimenticola symbionts… What efforts were made to obtain publicly available genomes that could resolve the deeper time events? Both of your outgroups (Allochromatium and Vibrio) contain nif genes, so perhaps including outgroups lacking nif might help? IMO, understanding the ancestral state for this whole clade would strengthen your claims substantially. This would allow to contextualize the nif-absent clades as either anciently lacking these genes or recently losing them. If they were recent losses, then the reacquisition of the genes by HGT might be more likely (due to co-evolved molecular networks).

Unless you have functional data for the lucinids and their symbionts, please tone down your functional claims. You couch statements well in the abstract, e.g., "*only those from the Caribbean had the genetic potential for nitrogen fixation*". However, in the main text there are many statements that go too far given that you only produced genomic data for this paper. I do not see references to physiological, metabolomic, etc. experiments to support these claims. For example:

> Lines 413-415: "*Specifically, TEP symbionts were able to synthesize gammapolyglutamate (Table I), a storage compound produced by bacteria during nutrient limitation [34].*"
>
> > The genomes predict that TEP symbionts can synthesize gammapolyglutamate - you don't show this directly. Please tone down the functional language.
>
> Lines 467-468: "*The ability to fix nitrogen was ubiquitous in the Ca. T. endolucinida clades from the oligotrophic waters of the Caribbean and Hawaii but absent in the TEP and Cape Verde clades…*"
>
> > Again, nitrogen fixation capabilities are predicted by the genomes. You do not demonstrate that they are actually doing this in vivo or in situ.

Lines 546-548: "*Paired-end Illumina sequencing produced reads of 150bp or 250 bp in length (SI Table). Illumina sequencing generated a minimum of 3,000,000 reads per sample.*"

> Read counts nor alignment coverage are in SI or any of the other tables.

Lines 572-547: "*Bins that were determined to be 90% or more complete and less than 10% contaminated post refinement were considered to be high-quality MAGs [64].*"

Table SII contains MAGs with scores outside of these criteria (e.g., I see some with completeness <80% and others with contamination >10-20%). Were they filtered out of the analysis?

Questions about the ancestral nifHDKT state reconstruction:

Lines 634-639: Is it valid to dereplicate MAGs differently across the tree to achieve a specific topology? I would think that choosing one value that performs well for all taxa would be a better, less biased choice.

Lines 651-652: "*MAGs that did not contain the full set of genes were excluded from the analysis.*"

How many were there? Were they randomly distributed or localized to some clade(s)?

Lines 653-654: "*A tree was inferred from each alignment to ensure that the genes have evolved together.*" Your nifHDKT phylogeny requires shared ancestries, however, your data are difficult to follow:

S1 Figure: It is very difficult for the reader to tell if these trees are the same because of the many tips and lack of labels. If you could calculate the percent concordance among trees and overlay the trees or align their tips in the figure, the reader could see how these data support your claims. As it stands, I am not going to spend the time looking at this figure and aligning tips to validate your claim.
