# Supplementary material for: Adaptations to nitrogen availability drive ecological divergence of chemosynthetic symbionts
Source: PLoS Genet. 2024 May 31;20(5):e1011295. doi: 10.1371/journal.pgen.1011295 (PMC11168628; doi:10.1371/journal.pgen.1011295)
Supplement: Attachment — Submitted filename: Response_to_reviewers_20240405.docx [file pgen.1011295.s015.docx]

Dear Editors,

We would like to express our sincere gratitude for the thorough evaluation of our manuscript at the editorial level and by independent reviewers. Your insightful comments and constructive feedback have been invaluable in guiding us through the revision process.

We have taken careful note of each point raised by the reviewers and have diligently addressed them in the revised version of our manuscript. A detailed point-by-point response has been included to document the specific changes and clarifications made in accordance with the reviewers' suggestions. Line numbers correspond to the clean copy of the revised manuscript.

We greatly appreciate the time and effort you and the reviewers have dedicated to evaluating our work. Your feedback has not only strengthened the quality of our manuscript but has also helped us refine our research findings. We are confident that the revisions have enhanced the clarity and coherence of our manuscript, and we are enthusiastic about the opportunity to resubmit it for your consideration.

Once again, we extend our sincere thanks for your valuable feedback and the opportunity to re-submit it to PLoS Genetics.

Yours sincerely,

Laetitia Wilkins, on behalf of all the co-authors

## REVIEWER 1

#### Overview:

Beautiful work showing horizontal gene transfer in an endosymbiosis that underwent dramatic divergence when the isthmus of Panama formed. The host speciated due to vicariance and the endosymbiont on the oligotrophic Caribbean side was swapped for a N-fixing strain that had acquired its N-fixing genes by HGT prior to the association. I think this work is important and well-done. However, I have a few concerns about the completeness of the assemblies, given the reliance of many claims on “gene absence”. Hopefully most of these concerns can be addressed by more transparent presentations of assembly completeness and coverage (as described below). I am also concerned about the functional claims that are made given that these are genome assembly-based predictions and no nitrogen-fixation, or other physiological, data were obtained or cited. Lastly, I think the authors could have done more due diligence in resolving the ancestral state of the Ca. Thiodiazotropha + Sedimenticola most recent common ancestor. Overall, I think these issues can be addressed in a short time frame, so I recommend major revisions.

We would like to extend our heartfelt gratitude to Reviewer #1 for their dedicated time and effort in reviewing our manuscript. We understand that reviewing manuscripts is a voluntary and time-consuming task, and we sincerely appreciate their commitment to providing thoughtful feedback.

Reviewer #1's encouraging words and constructive feedback have been immensely valuable to us. Their insights have contributed significantly to the improvement of our manuscript, and we are grateful for their thorough evaluation and helpful suggestions. Please note that the line numbers correspond to the clean copy of the revised manuscript.

#### Minor Comments:

Figure 1D: the top axis labels are flip-flopped (symbiont clade = purple or green / host genus = violet or pink).

We thank Reviewer 1 for pointing out this error. Figure 1 has been modified accordingly.

Figure 3B: Since many of your claims rely on ecology and geography it would help the reader to have the other samples in the tree (that you didn’t collect/sequence) color coded by geography.

Figure 3A and Figure 3B have been extensively modified to inform the geographic distribution of the different clades in the most intuitive way possible. In addition, to improve the clarity of the section where we compare recombination rates between globally distributed clades, we have included a new supplementary figure (S5 Figure). This figure shows the geographic origin of only those samples that contained globally distributed symbionts, differentiated by the study in which they were obtained.

Line 443: typo in the word “closely” - “the Pegophyseminae symbionts “PEGO” or an unsampled closel related lineage.”

Typo corrected (L455).

Lines 479-480: “This suggests there is no barrier to gene flow between geographically separated populations of Ca. T. endolucinda”

There might be some barriers that could be quantified with a proper admixture demographic analysis. It is safer (in terms of accuracy) to say “low barriers to gene flow”.

The phrasing has been changed to “low barriers” (L496-497).

Lines 548-549: “Samples ctemesantah004, cteimcahuit014, and cteimbast119 were also sequenced using PacBio Sequel II long-read technology.”

Why only these samples? I can’t find an explanation for the choice anywhere in the manuscript.

Samples ctemesantah004, cteimcahuit014, and cteimbast119 each contained different symbiont lineages, *Ca*. T. endolucinida TEP, *Ca*. T. taylori, and *Ca*. T. fergusoni, respectively. This has been determined previously based on Illumina short-read sequencing. These samples were selected in an attempt to obtain circularised genomes from the more prevalent symbiont taxa. We have changed those lines of text in the Methods section to clarify the rationale behind the use of PacBio sequencing for these samples (L581-584).
“Samples ctemesantah004, cteimcahuit014, and cteimbast119, for which Illumina MAGs of the symbiont lineages Ca. T. endolucinida TEP, Ca. T. taylori, and Ca. T. fergusoni had been respectively recovered, were selected for long-read sequencing with the goal of obtaining circularized symbiont MAGs of some of the most prevalent clades present across the Isthmus”

#### Major Comments:

Line 610: “ANI values were calculated with fastANI v1.1”

Are all ANI values based on genome-wide comparisons? Did you only include “core” regions? Please state what ANI was calculated from in the text (I didn’t see it elsewhere either).

The fastANI approach is widely used and relies on alignment-free sequence mapping to compare pairwise sequence identity across entire genomes. The mean ANI values from fastANI are computed from bi-directional searches of fragments from each genome, which inherently requires the fragments of the query and reference genomes to be shared. This approach does not require a pre-processing step to identify shared or core regions through sequence alignment.

Figure 1 legend (line 188) and Figure 2 legend (line 300): Why was the plant protein model matrix (Q.plant+F+I+G4) used for bacterial symbiont tree inference? See <http://www.iqtree.org/doc/Substitution-Models>.

IQ- TREE was always run with the new substitution model detection algorithm (-m MFP ) to determine the model that best fit our data:

L624-626: "A maximum likelihood phylogenomic tree was inferred using IQ-Tree v2.2.2.1 [72–74] with auto substitution model detection, 1,000 ultrafast bootstrap (UFB) replicates and 1,000 samples for SH-aLRT branch testing"

In this case, the IQ-TREE model finder repeatedly selected the plant protein model. In the link to the IQ-TREE documentation provided by Reviewer 1, it is also clear that bacteria or archaea-specific Amino-acid exchange rate matrices are not available for the automatic model finder to select. Finally, there is no inherent problem in fitting the Q.plant model to our data. It is possible that the factors driving selection across the lucinid symbiont genomes might result in the symbiont proteins evolving in ways that best fit Q.plant, amongst all the pre-estimated models that are available in the IQ-TREE software package. Similar outcomes have been observed by other users of IQ-TREE in the past (See <https://groups.google.com/g/iqtree/c/SOAZzZBqBYc>).

So many of your functional hypotheses rely on the absence of particular genes (Figures 2-4). However, I don’t have a good sense for how complete these MAGs are by the time you start discussing the lack of form II rubisco in Ca. T. fergusoni on lines 224-225 and the absence of nitrogen pathways in TEP MAGs on lines 231-234. Annotated genes may be missing due to broken, incomplete assemblies. You have a very clear signal across samples in Figure 2, so I think this signal is not due to some technical artifact. But it would help the reader have confidence in your subsequent claims to present coverage and completeness statistics upfront.

We have included coverage and completeness statistics for all the MAGs we generated in SII_Table (methods described in L596-599). There were no obvious differences that would suggest our findings were influenced by technical artifacts. The mean coverage for both Caribbean and TEP MAGs was generally high, with average values of 118.57 and 109.66, respectively. Additionally, we didn’t observe big differences in the average completeness (98.73% for Caribbean and 98.81% for TEP) or contamination (0.57% for Caribbean and 0.82% for TEP) of the MAGs included in the analyses.

Finally, in addition to these quality control data, we were also able to obtain circularised genomes of *Ca*. T. fergusoni from two separate host individuals and a circularised genome of *Ca* T. endolucinida TEP. These lines of evidence support the conclusion that nitrogenase and form II rubisco genes were not present in the genomes of *Ca* T. endolucinida TEP and *Ca*. T. fergusoni, respectively.

You do seem to try to account for an ascertainment bias - that the TEP nitrogen genes weren’t annotated - when you state (on lines 245-247): “no hits were reported from the hmmer search search of the Pfam nitrogenase model against the TEP metagenome assemblies, supporting the lack of nitrogen fixation potential in the TEP symbiont genomes”. But, what about in the unassembled reads? Were these assemblies more fragmented than the Caribbean MAGs? If so, maybe the nitrogen genes were in the regions that could not assemble. I need some evidence that this is not the case.

We have now included the statistics from aligning the raw metagenomic reads to the key functional nitrogenase genes NifDHKT (Dataset S5). The corresponding results for the HMM search against the Caribbean and TEP assemblies are also displayed in Dataset S5. These data support our conclusions that the nitrogenase genes were absent in the genomes of the TEP clade.

Layering assembly quality information on Table 1 could help contextualize the patterns of incomplete presence/absence. I.e., are those near, but not total fixations for some KOfam terms among CaribbeanMAGs due to the same samples, and did those samples produce worse assemblies?

We have now included raw assembly and MAG quality information (Tables SI and SII). There were no obvious differences in assembly quality between symbiont MAGs originating from the Caribbean and TEP. For example, the average N50 for Caribbean and TEP assemblies was 2.75E+05 bp and 2.41E+05 bp, respectively. In addition, the average L50 was lower for TEP assemblies (357.26) than for Caribbean assemblies (431.92), indicating less fragmentation of the former.

Lines 625-628: “To confirm the lack of nitrogen fixation potential in TEP symbionts’ genomes and ensure that the genes were not missed in the binning process, the proteins were directly predicted from all TEP metagenome assemblies with Prodigal v2.6.3 [80].”

This will still miss genes that are on unassembled or fragmented contigs. Attempting to “fish” the genes out by read mapping would enable you to look for these genes with less assembly-based bias.

We have carried out read mapping to the functional nitrogenase genes *NifDHKT* but this did not result in any hits in most of the TEP symbiont genomes (Dataset S5). A few metagenomes returned a small number of reads mapping to the functional genes, but these reads are more likely to be contaminants as the number of reads mapped were orders of magnitude below the average coverage of reads mapping across the respective MAGs (Dataset S5 and SII_Table). These findings are now reported in the Results section (L246-249).

Lines 459-462: Indeed, I was thinking that including a few more divergent outgroup taxa could enable you to determine the nif content state for the last common ancestor of the Ca. Thiodiazotropha and Sedimenticola symbionts… What efforts were made to obtain publicly available genomes that could resolve the deeper time events? Both of your outgroups (Allochromatium and Vibrio) contain nif genes, so perhaps including outgroups

lacking nif might help? IMO, understanding the ancestral state for this whole clade would strengthen your claims substantially. This would allow to contextualize the nif-absent clades as either anciently lacking these genes or recently losing them. If they were recent losses, then the reacquisition of the genes by HGT might be more likely (due to co-evolved molecular networks).

We thank the reviewer for the helpful suggestions for improving this aspect of our study. We have since redone the ancestral reconstruction analysis with 377 additional publicly available genomes and MAGs representing a diverse collection of Chromaticaeae and Sedimenticolaceae obtained from GTDB (in methods sections L664-669). The results of this analysis have allowed us to resolve the ancestral state of nitrogen fixation potential in the *Ca*. Thiodiazotropha and *Sedimenticola* symbiont genera. These new findings are described in Figure 4, which is a pruned version of the full reconstruction. The full reconstruction of the order Chromatiales, in turn, can be found in S3 Figure. These results are now described in L348-356.

Unless you have functional data for the lucinids and their symbionts, please tone down your functional claims. You couch statements well in the abstract, e.g., “only those from the Caribbean had the genetic potential for nitrogen fixation”. However, in the main text there are many statements that go too far given that you only produced genomic data for this paper. I do not see references to physiological, metabolomic, etc. experiments to support these claims. For example:

Lines 413-415: “Specifically, TEP symbionts were able to synthesize gammapolyglutamate (Table I), a storage compound produced by bacteria during nutrient limitation [34].”

The genomes predict that TEP symbionts can synthesize gammapolyglutamate - you don’t show this directly. Please tone down the functional language.

We have rephrased this sentence with more conservative language. It now reads (L421-422): “Specifically, TEP symbionts had the genetic potential for synthesizing gammapolyglutamate (Table I), a storage compound produced by bacteria during nutrient limitation [34].”

Lines 467-468: “The ability to fix nitrogen was ubiquitous in the Ca. T. endolucinida clades from the oligotrophic waters of the Caribbean and Hawaii but absent in the TEP and Cape Verde clades…”

Again, nitrogen fixation capabilities are predicted by the genomes. You do not demonstrate that they are actually doing this in vivo or in situ.

We have rephrased this sentence with more conservative language. It now reads (L484-487): “The genes for fixing nitrogen were ubiquitous in the genomes of *Ca.* T. endolucinida clades from the oligotrophic waters of the Caribbean and Hawaii but absent in the TEP and Cape Verde clades originating from upwelling regions characterized by sporadically high levels of bioavailable nitrogen [38,45].”

Lines 546-548: “Paired-end Illumina sequencing produced reads of 150bp or 250 bp in length (SI Table). Illumina sequencing generated a minimum of 3,000,000 reads per sample.”

Read counts nor alignment coverage are in SI or any of the other tables.

We thank Reviewer 1 for pointing out this data omission and have included additional data for the raw assemblies and MAG coverage for each sample in Tables SI-II.

Lines 572-547: “Bins that were determined to be 90% or more complete and less than 10% contaminated post refinement were considered to be high-quality MAGs [64].”

SII_Table contains MAGs with scores outside of these criteria (e.g., I see some with completeness <80% and others with contamination >10-20%). Were they filtered out of the analysis?

Low and medium quality publicly-available MAGs were only included in the phylogenomic analysis (see Figure 3B), but these low quality MAGs were not used for any comparative genomics analysis. Only high quality MAGs (>90% complete and <10% contaminated) were used in the final analyses as stated in L597-601: "Bins that were determined to be 90% or more complete and less than 10% contaminated post refinement were considered to be high-quality MAGs [64]. Only high-quality MAGs classified as Gammaproteobacteria by the GTDB-Tk v2.1.1 (Genome Taxonomy Database Toolkit) classify workflow [65–68] were used for further analyses." We have also included columns in SII Table which state clearly which MAGs at each step of our analyses.

Questions about the ancestral nifHDKT state reconstruction:

Lines 634-639: Is it valid to dereplicate MAGs differently across the tree to achieve a specific topology? I would think that choosing one value that performs well for all taxa would be a better, less biased choice.

We have redone the ancestral reconstruction analysis with MAGs that were de-replicated at a 99.5% ANI threshold (Figure 4, methods in L667-669). The list of MAGs used for each analysis is now available in SII Table and, specifically, the resulting MAGs after dereplicating in column “After_dREP_99.5”.

Lines 651-652: “MAGs that did not contain the full set of genes were excluded from the analysis.”

How many were there? Were they randomly distributed or localized to some clade(s)?

We excluded a total of seven *Ca.* Thiodiazotropha MAGs, from four different clades (details were added to “Ancestral reconstruction” column of SII_Table). Most of these belonged to the *Ca.* T. taylori clade, which nonetheless remains highly represented in the final tree.

The following MAGs were removed after the de-replication step:

| **MAG_ID** | **Clade** |
| --- | --- |
| Codakia_orbicularis_STRI_SYM1_codorstrist081run1 | *Ca*.T.endolucinidaCAR |
| Cryptophysema_vesicula_Okinawa_SYM_L066 | PEGO |
| Loripes_orbiculatus_FetoviaItaly_SYM1_ELBAC10 | *Ca*.T.lotti |
| Callucina_winkworthi_Mauritius_SYMTRANSOCEAN_L082 | *Ca.*T.taylori |
| Clathrolucina_costata_BocasdelDrago_SYMTRANSOCEAN_clacodrago038 | *Ca.*T.taylori |
| Clathrolucina_costata_Cristobal_SYMTRANSOCEAN_clacocristo008 | *Ca.*T.taylori |
| Clathrolucina_costata_PuntaPuebla_SYMTRANSOCEAN_clacopuebla005 | *Ca*.T.taylori |

Lines 653-654: “A tree was inferred from each alignment to ensure that the genes have evolved together.” Your nifHDKT phylogeny requires shared ancestries, however, your data are difficult to follow:

S1 Figure: It is very difficult for the reader to tell if these trees are the same because of the many tips and lack of labels. If you could calculate the percent concordance among trees and overlay the trees or align their tips in the figure, the reader could see how these data support your claims. As it stands, I am not going to spend the time looking at this figure and aligning tips to validate your claim.

We apologize for the poor resolution in the supplementary figures. S1 Figure has been updated to improve legibility.

## REVIEWER 2

#### Overview:

The environment is an important driving force for the evolution of bacterial symbionts, especially for the horizontally transferred endosymbionts. In the current study, the authors forced on the sister species of two lucinid genera, Codakia and Ctena, distributed in distinct environmental conditions in the Tropical Eastern Pacific and Caribbean to reveal the influence of the environment in the genomic and metabolic divergence of endosymbionts. Besides, the authors also revealed convergent evolution of nitrogen fixation genes in different evolutionary lineages under nutrient-poor conditions by comparing different lucinid symbionts across the globe. Overall, the manuscript is well-organized, and the results are thoroughly discussed. The authors provided valuable resources on the symbionts of Lucinidae and contributed to the understanding of the evolution and adaption of marine symbiosis. Therefore, I would like to suggest that the manuscript be published after minor revision.

We would like to extend our sincere gratitude to Reviewer #2 for their insightful comments and constructive feedback on our manuscript. Their recognition of the importance of environmental factors in the evolution of bacterial symbionts, along with their positive assessment of our study's organization and discussion, is greatly appreciated.

We were very encouraged by Reviewer #2's recommendation for publication with minor revisions. Their contributions have undoubtedly strengthened the quality and impact of our manuscript.

Please note that the line numbers correspond to the clean copy of the revised manuscript.

#### Detailed comments to the authors:

Lines 174-178, different numbers of MAGs were assembled from the individuals of the two lucinid genera, Codakia and Ctena, and significantly more MAGs were assembled from Ctena individuals. Are the differences in the number of assembled genomes related to the sequencing methods or to host species?

Differences in the number of MAGs assembled from each host species do not appear to be a technical artifact due to differences in sequencing coverage or technology as all samples were to similar depths using the same Illumina platform (SI_Table). One potential explanation could be sampling bias, as *Ctena* individuals were more abundant in the environment and so we are more likely to see the full diversity of symbionts associated with these species.

In Fig. 1B-C and the relative text in the part of results, I did not find the descriptions of the host species and their corresponding symbiont clades, although they can be found in the S1_table. This information will help evaluate the specificity between the host species and symbiont clades. Therefore, I suggested making it clear about the result in the main text.

We thank Reviewer 2 for this suggestion. We have added the corresponding host genera information to Figure 1C and to the results section in L216-219:

“MAGs classified as Ca. T. fergusoni, Ca. T. endolucinida CAR and Ca. T. endolucinida TEP were recovered from both Codakia and Ctena specimens, while Ca. T. taylori, Ca. T. boucheti and Ca. T. larai MAGs were only recovered from Ctena specimens (Fig. 1C and D).”

In Fig. 1D, the host genus and the symbiont clade were incorrectly annotated at the top of the heatmap.

We thank Reviewer 1 for pointing out this error. Figure 1 has been modified accordingly.

Line 222, only three symbiont clades were found in the S2 Dataset, the data of other clades were missed in the table.

Sup. Dataset 2 was originally intended to describe the new symbiont clades. We have now included the metabolic reconstruction of the previously described clades to facilitate comparison with the newly described clades. A more complete and revised metabolic reconstruction of these clades can be found in the referenced papers: Osvatic 2021 and 2023.

Line 394, I do not fully agree with the conclusion that “symbiont choice was driven by geographic location rather than host species”. The two host species in genera Codakia hold two clades of Ca.T.endolucinida which is phylogenetically close and has higher ANI values. Besides, the number of symbiotic bacteria obtained from the two lucinid genera was different. In particular, in Ctena individuals, three different clades of symbiotic bacteria were obtained. Are these bacteria coexisted in the gill cells of the host? Are there any previous studies? The environment is responsible for the differentiation of endosymbionts, but the host species is also relevant because the establishment of endosymbiosis requires complex molecular mechanisms in the host cells.

We agree that the host is naturally relevant and important in the establishment of the symbiosis. We have toned down the language by removing the sentence “symbiont choice was driven by geographic location rather than host species” (L400-404). It now reads:
“We observed multiple instances where different host species of the *Codakia* and *Ctena* genera on the same side of the Isthmus share the same symbiont groups. This is consistent with horizontal symbiont acquisition from the environment and indicates that the environment is a key factor influencing symbiont selection and distribution.”

Although we recovered MAGs of *Ca.* T. taylori, larai, and boucheti only from *Ctena* specimens, we refrain from discussing patterns in host fidelity and promiscuity because the substantial over-sampling of *Ctena* (108 MAGs) compared to *Codakia* (14 MAGs) makes it difficult to exclude the possibility that these patterns result from sampling bias.

Unfortunately, we were unable to obtain samples of these *Ctena* gills for FISH and microscopic imaging, so we have opted not to discuss this pattern in further detail. However, it has been reported that different symbiont species coexist in the same host but do not share the same bacteriocytes (Osvatic et al. 2021).

Lines 522-541, the sample collection part is unclear to me. The authors listed many samples, but it is unclear which was used for sequencing in the manuscript. I think it is better to clearly describe which individuals were newly acquired or sequenced in the current study and which were previously obtained by the authors or other studies, and references should be listed if they have been published.

Additionally, S1_table has a similar problem. All the data used in the manuscript were put together in the table. The distinction between newly generated data and data from the previous studies is not clear. In the column of the Paper, the references to the previous studies should be correctly cited.

We have chosen not to list the sources of each species/sample in the manuscript for clarity and conciseness due to the sheer number of different samples/species included in the study. However, we understand the reviewer's concern and SI_Table has been updated to include all the metagenome metadata of the samples collected and sequenced for this study. Also, all publicly available MAGs that we used are now clearly labeled in the "Source" column of SII_Table together with their corresponding accession number. Finally, we have also added a symbology to Fig. 3B to show in the tree which clades are a contribution of this study.
